# Role of Mentzer index for differentiating iron deficiency anemia and beta thalassemia trait in pregnant women

**DOI:** 10.12669/pjms.38.4.4635

**Published:** 2022

**Authors:** Shagufta Tabassum, Mehnaz Khakwani, Asiya Fayyaz, Nadia Taj

**Affiliations:** 1 Dr. Shagufta Tabassum, FCPS, Department of Obstetrics & Gynaecology, Nishtar Medical University, Multan, Pakistan; 2 Dr. Mehnaz Khakwani, FCPS, Department of Obstetrics & Gynaecology, Nishtar Medical University, Multan, Pakistan; 3 Dr. Asiya Fayyaz, FCPS, Department of Obstetrics & Gynaecology, Nishtar Medical University, Multan, Pakistan; 4 Dr. Nadia Taj, FCPS, Department of Obstetrics & Gynaecology, Nishtar Medical University, Multan, Pakistan

**Keywords:** Iron deficiency anemia, Beta thalassemia, Mentzer Index

## Abstract

**Objective:**

To observe the role of Mentzer index for differentiating iron deficiency anemia (IDA) and beta thalassemia trait (β TT) in pregnant women.

**Methods:**

This cross-sectional study was conducted in Gynaecology & Obstetrics Department of Nishtar Medical University from October 2020 to March 2021. Non-consecutive sampling was applied. A total of 100 antenatal ladies with hemoglobin <11 gm/dl were included. Their complete blood counts were checked and Mentzer Index was calculated. Mentzer Index <13 points to diagnosis of β TT and >13 indicates IDA. The diagnoses were confirmed by serum iron studies and Hb electrophoresis. The sensitivity and specificity of Mentzer Index for both causes of microcytic hypochromic anemia was calculated.

**Results:**

Out of total 100 patients with microcytic hypochromic anemia, 87 had Mentzer Index >13 and IDA was confirmed in 86 out of 87 cases. Thirteen cases had Mentzer Index <13 and β TT was confirmed in eight of them. Thus, Mentzer Index has a sensitivity and specificity of 91% & 83% for IDA and 83% & 91% for β TT.

**Conclusion:**

In this study, it was found that Mentzer Index can be used as a discriminatory test to differentiate between iron deficiency anemia and beta thalassemia trait. The high risk group can then be subjected to definitive diagnostic tests. This can result in better patient compliance and cost effectiveness.

## INTRODUCTION

Anemia in pregnancy is a global health concern^1^ encountered by obstetricians worldwide. However, it is much more prevalent in developing countries including Pakistan.^2^ Its incidence varies from 19% in developed countries to as high as 35-75% in developing countries.^3^ In Pakistan, anemia in pregnancy has high prevalence of about 42%^4^, so much so that prophylactic iron supplements are prescribed to almost all pregnant ladies. This anemia is most likely due to iron deficiency but may also be due to underlying hemoglobinopathy, most common of whom is beta thalassemia trait (β TT).

While iron supplements are very beneficial in iron deficiency anemia (IDA), they can be devastating if the anemia is due to thalassemia. Both of these types of anemia have more or less similar symptoms of shortness of breath, fatigue, dizziness and irritability. These symptoms are more marked in case of iron deficiency anemia while quite a number of persons with β TT are asymptomatic. In both conditions, the complete blood counts reveal the picture of microcytic hypochromic anemia.^5^ It necessitates specific tests to differentiate between the two conditions. Iron deficiency anemia is confirmed by iron studies and ferritin levels while β TT diagnosis needs estimation of HbA2 by hemoglobin electrophoresis. A level of HbA2 > 3.5% is considered pathognomonic of beta thalassemia.

Inability to differentiate between iron deficiency anemia and beta thalassemia trait on the basis of blood picture and non-affordability of tests like Hb electrophoresis and iron studies has led to the invention of indices using blood counts. The most commonly used is Mentzer Index, devised by Mentzer in 1973. In iron deficiency anemia, a smaller number of red blood cells are produced by bone marrow, resulting in low red blood cell (RBC) counts and low mean corpuscular volume (MCV). So the Mentzer Index is >13. In thalassemia, the number of RBCs produced is normal but due to defective beta globin chain, the red blood cells are smaller in size and more fragile, resulting in normal red blood cell count and low mean corpuscular volume. So the Mentzer Index is <13.

Thalassemia history dates back to almost a century when it was first described by Dr Thomas Cooley in children of Mediterranean origin.^6^ Without treatment, those having thalassemia major have severe anemia within first two years of life and shortened life expectancy.^7^ Those having βTT face mild anemia in later years of life and it is this class of thalassemia which present as antenatal population. The diagnosis of beta thalassemia is of utmost importance as iron treatment can lead to devastating sequelae including hepatic and cardiac damage due to iron overload. Also, the diagnosis of thalassemia can lead to partner’s hemoglobinopathy identification and prenatal diagnosis of offspring by chorionic villous sampling.

As regards thalassemia, worldwide its carrier rate is 3%^8^ while it is much more prevalent in South East Asia. In Pakistan, its carrier rate is 5-8%^9^ with consanguinity being the most common contributory factor. In a developing country like Pakistan, this genetic disorder poses a serious risk to health sector as frequent blood transfusions along with iron chelation are quite expensive which may not be affordable by all the affected people.^10^ Preventive strategies include carrier identification, genetic counseling and prenatal diagnosis. Carrier identification requires hemoglobin electrophoresis, which is again an expensive test, making it almost impossible to perform this test on whole population or even high risk groups. This necessitates some cost effective screening tests which can be applied on mass levels. Various screening tests are available to discriminate between IDA and βTT. These include Mentzer Index (MI), Green & King Index (G&K), Red cell distribution Index (RDWI), England & Fraser Index (E&F), Shine & Lal Index, Red Cell Distribution, and red blood cell count. Out of these, Mentzer Index is a likely and practicable choice as complete blood counts are performed as a preliminary test at booking antenatal visit and again at 28 weeks of gestation. Complete blood counts include hemoglobin along with mean corpuscular volume (MCV), mean corpuscular hemoglobin (MCH), mean corpuscular hemoglobin concentration (MCHC) and red blood cell counts. Mentzer Index is calculated as MCV(fl) / RBC count (millions per microliter); if the result is >13, IDA is the probable diagnosis while a value < 13 indicates to likelihood of beta cell thalassemia.^11^ Thus, those with Mentzer Index <13 can be subjected to estimation of HbA2 by hemoglobin electrophoresis for confirmation of βTT. This can be quite cost effective as only those with high probability of having beta thalassemia undergo the financial burden of hemoglobin electrophoresis.

Globally thalassemia is the most prevalent yet preventable genetic hematological disorder.^12^ Pakistan is one of those countries with the highest thalassemia burden.^13^ It is estimate that about 2.7 million blood collections are made in Pakistan annually, out of which one fourth are used for thalassemic patients.^14^ This is quite a large figure of blood transfusions for a developing and resource-constrained country like Pakistan. Keeping in view the worth of diagnosis of thalassemia, a study was conducted in anemic pregnant ladies to validate the role of Mentzer Index to differentiate between IDA and β TT by assessing simple parameters of complete blood counts.

## METHODS

This cross sectional study was conducted from October 2020 to March 2021, in Obstetrics and Gynaecology Department of Nishtar Medical University in Multan city of Pakistan. Non probability consecutive sampling was used. All those antenatal cases who met inclusion criteria of hemoglobin < 11 gm/dl were included while those having other known causes of anemia like autoimmune hemolytic anemia, aplastic anemia, anemia of chronic illness or blood transfusion during last month were excluded.

Ethical clearance was taken from the Institutional Ethical Committee (Reference letter number 4411; dated March 11, 2021) to conduct this study. Informed consent was taken from each patient before inclusion.

The patients were briefed about the objective of the study, ensuring them confidentiality of the information provided and making them confident that there will be no harm to them while taking part in this study. Those fulfilling the inclusion criteria were registered and all the information was noted on the proforma. Demographics of the study subjects were collected including age, address, educational status, gestational age, gravidity, eating habits, family history of thalassemia. The symptoms were also noted including shortness of breath, fatigue, dizziness, irritability, palpitations and anorexia. Their blood sample in Ethylenediamine tetraacetic acid (EDTA) was sent for complete blood counts. All the subjects were subjected to measurement of serum ferritin level, serum iron level and HbA2 level by Hb electrophoresis.


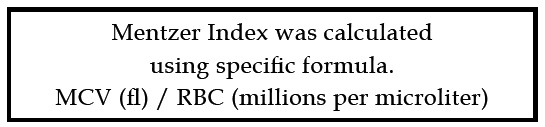


All the data were analyzed using Statistical package for Social Services (SPSS) using version 20. Percentages and frequencies were calculated. P value equal to or less than 0.5 was considered to be significant. Sensitivity and specificity of Mentzer Index was calculated for both iron deficiency anemia and beta thalassemia trait.


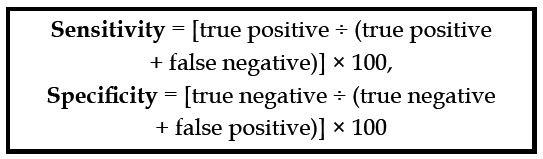


## RESULTS

In this study, 100 antenatal patients with microcytic hypochromic anemia were taken into account. Their mean age was 25 years (+/-3 years). Mean gravidity was three. Most of them were urban residents. This corresponds with the fact that Nishtar Medical Hospital is a city-cited medical consultation center. Most (62%) had unsatisfactory intake of iron containing food items. Only one patient confessed family history of thalassemia.

Their symptomology was evaluated and revealed that most common symptom of iron deficiency anemia was fatigue (70%) followed by palpitations (40%). Other symptoms encountered in iron deficiency anemia were breathlessness (21%), irritability (16%) and anorexia (13%) while 20% were asymptomatic. Contrarily, 60% were asymptomatic in βTT group. In the remaining persons with βTT, symptomatology involved fatigue (34%), irritability (21%) and breathlessness (12%).Ninety four percent of patients were suffering from Iron Deficiency Anemia while Beta Thalassemia Trait was found in six percent of cases.

Mentzer Index was calculated for all the 100 people. It was found to be > 13 in 87 cases and <13 in 13 cases. These 100 subjects were subjected to serum iron studies, serum ferritin and estimation of HbA2 level estimation by Hb electrophoresis. (Table-I)

**Table-I:** Mentzer Index in cases of iron deficiency anemia and beta thalassemia trait.

	Mentzer Index >13	Mentzer Index <13
Iron deficiency anemia	86	8
Beta thalassemia trait	1	5

In medical diagnosis, test sensitivity is the ability of a test to correctly identify those with the disease (true positive rate), whereas test specificity is the ability of the test to correctly identify those without the disease (true negative rate). Table-II reveals that Mentzer Index is more reliable in diagnosing true positive cases of iron deficiency anemia with a sensitivity of 91% while it is more effective in detecting true negative cases of βTT with a specificity of 91%.

**Table-II:** Sensitivity and specificity of Mentzer Index for iron deficiency anemia and beta thalassemia trait.

	Sensitivity	Specificity
Iron deficiency anemia	91%	83%
Beta thalassemia trait	83%	91%

## DISCUSSION

In current study, out of 100 subjects, 93% were diagnosed to be having IDA and 7% were labeled as having βTT. The prevalence of thalassemia trait in the current study is close to that estimated by Ahmed et al (5.6%).^15^ It also closely mimics to that calculated in Karachi in a general population survey.^16^ However, it is quite higher than the worldwide prevalence of βTT which is estimated to be 1.5%.^17^ The prevalence of βTT in Pakistan is higher than that observed worldwide. It is due to the fact that more than 50% of beta thalassemia carriers are found in Southeast Asia^18^ and Pakistan is also situated in this geographical location. Also consanguineous marriages play the leading role for these high figures in Pakistan where religious, cultural and economic reasons still lead to consanguineous marriages.

Various indices have been used in an attempt to differentiate between iron deficiency anemia(IDA) and beta thalassemia by using simple blood counts parameters but none has so far been found to be 100% sensitive and 100% specific. It is found that Mentzer Index has got maximum sensitivity and specificity among all relevant indices.^19^ In this study also, it was found that Mentzer Index can be used as a screening test to differentiate between IDA and βTT. This was also verified by Munir AH et al in their study.^20^ Those with Mentzer Index > 13 are most probably having IDA with a sensitivity of 91% and having non probability of beta thalassemia with a specificity of 91%. Contrarily, those antenatal women with microcytic hypochromic anemia, whose Mentzer Index is <13 are more probably having βTT with a sensitivity of 83% and having non-probability of IDA with a specificity of 83%. Thus, Mentzer Index is more reliable in diagnosing IDA and excluding βTT. This was also concluded in a study conducted by Bose S et al. in India.^19^ Similar results were reached by D Lawrie in his study conducted in South Africa.^21^ Alam et al. also found more or less similar results^18^.

When compared with other hematological indices used to differentiate between the two important causes of microcytic hypochromic anemia, Mentzer index has proved to be most reliable in a study conducted by Vehapoglu A et al^22^. Similar results were also documented by Sundh A et al^23^.

In brief, important differential diagnoses of microcytic hypochromic anemia are iron deficiency anemia and beta thalassemia. Both of these necessitate specific tests for confirmation which are not only expensive but also not easily available. This necessitates some reliable screening test or index to spotlight high risk group, which can be offered confirmatory tests to reach at specific diagnosis. The Mentzer Index can be used as a screening test to help differentiate between iron deficiency anemia and beta thalassemia.

### Limitations of study:

The sample size should be large and other red cell indices should also be included for a better comparison of various tests available to differentiate iron deficiency anemia from beta thalassemia trait.

## CONCLUSION

The demarcation between iron deficiency anemia and beta thalassemia in anemic antenatal women poses a challenge to clinicians as the diagnostic tests are not affordable by most of people. The Mentzer Index can be used as a screening test with a sensitivity and specificity of 91% & 83% for iron deficiency anemia and 83% & 91% for beta thalassemia. Those with Mentzer Index < 13 can be subjected to Hb electrophoresis for confirmation.

### Authors’ Contribution:

**ST:** Conceived, designed, manuscript writing, statistical analysis, is responsible for integrity of research.

**AF**, **NT:** Did data collection and editing of manuscript.

**MK:** Did review and final approval of manuscript.
